# Correction: Biomimetic proteoglycan nanoparticles for growth factor immobilization and delivery

**DOI:** 10.1039/d5bm90019k

**Published:** 2025-03-03

**Authors:** Nooshin Zandi, Ebrahim Mostafavi, Mohammad Ali Shokrgozar, Elnaz Tamjid, Thomas J. Webster, Nasim Annabi, Abdolreza Simchi

**Affiliations:** a Institute for Nanoscience and Nanotechnology, Sharif University of Technology P.O. Box 11365-11155 Tehran Iran simchi@sharif.edu +98 (21) 6616; b Department of Chemical Engineering, Northeastern University Boston 02115 USA; c National Cell Bank Department, Pasteur Institute of Iran Tehran 13164 Iran; d Department of Nanobiotechnology, Faculty of Biological Sciences Tarbiat Modares University P.O. Box 14115-175 Tehran Iran; e Department of Chemical and Biomolecular Engineering, University of California – Los Angeles Los Angeles California 90095 USA nannabi@UCLA.edu +1 (310) 267-5927; f Center for Minimally Invasive Therapeutics (C-MIT), California NanoSystems Institute (CNSI), University of California – Los Angeles 570 Westwood Plaza Los Angeles CA 90095 USA; g Harvard-MIT Division of Health Sciences and Technology, Massachusetts Institute of Technology Cambridge MA 02139 USA; h Department of Materials Science and Engineering, Sharif University of Technology P.O. Box 11365-11155 Tehran Iran

## Abstract

Correction for ‘Biomimetic proteoglycan nanoparticles for growth factor immobilization and delivery’ by Nooshin Zandi *et al.*, *Biomater. Sci.*, 2020, **8**, 1127–1136, https://doi.org/10.1039/C9BM00668K.

The authors regret the representative images for the Control group in Fig. 4a, and GT-PLL Day 14 and Free VEGF Day 7 in Fig. 5a were incorrectly displayed in the original manuscript. The correct versions of Fig. 4 and 5 are as shown below.

**Fig. 4:**
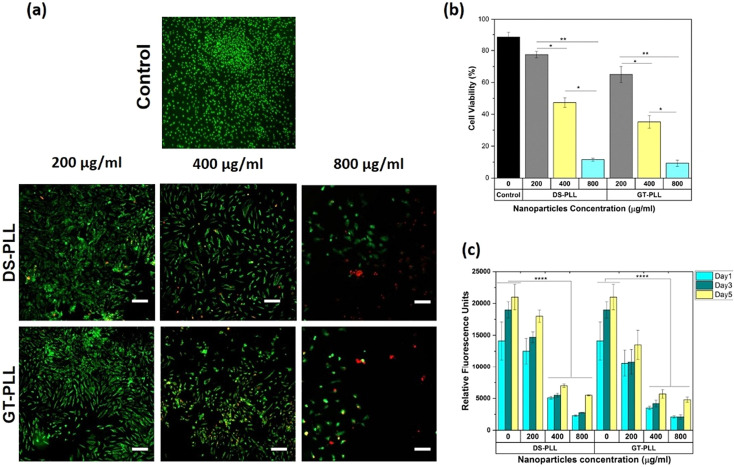
*In vitro* cytocompatibility of the polyelectrolyte NPs against HS-5 cells. (a) Representative live/dead stained images, indicating the effect of PCN (DS-PLL and GT-PLL) dose on the cell viability at day 3 post seeding. The concentration of NPs increases from 200 to 800 μg mL^−1^ from left to right. Cytotoxicity was observed for the cells treated with NPs at concentrations >400 μg mL^−1^. (b) Quantification of cell viability after 24 h of incubation. (c) Quantification of metabolic activity of hBMSCs based on relative fluorescence units (RFU) at different incubation times (1, 3 and 5 days post seeding). Scale bars: 100 μm. Results are presented as the mean ± STD with at least three replicates per group. The significance levels are shown as *p* < 0.05 (*), *p* < 0.01 (**), and *p* < 0.0001 (****) for *n* = 3.

**Fig. 5:**
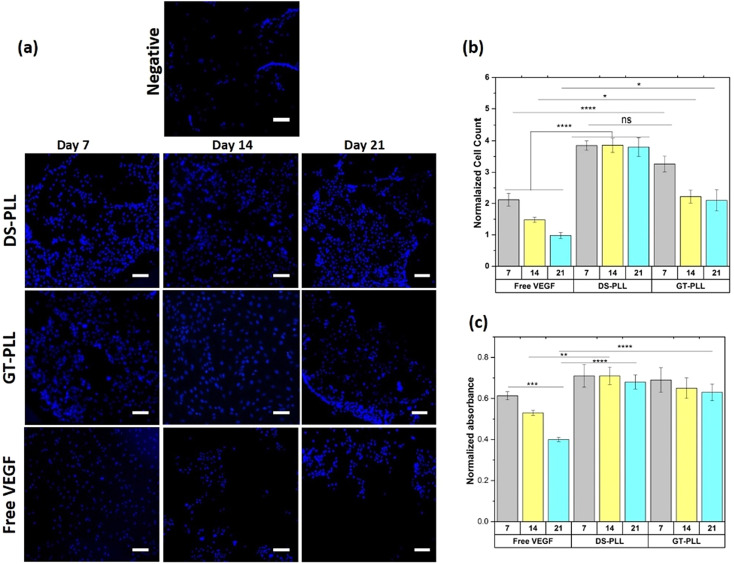
Cell response to VEGF and pre-conditioned VEGF-loaded PCNs through mitogenic and metabolic measurements. (a) Representative fluorescence images of HUVEC nuclei stained with DAPI after 2 days of culture with no treatment (negative control), VEGF-loaded PCNs (DS-PLL and GT-PLL), and free VEGF. Pre-conditioning time does not show a significant influence on mitogenic activity, but the treatments exhibit significant effects. (b) Quantification of VEGF mitogenic activity after 2 days of HUVEC culture for VEGF-loaded PCNs and free VEGF. The numbers represent cell counts normalized to cell counts from the negative control. (c) The quantification of metabolic activity after 2 days of culture with VEGF-loaded PCNs and free VEGF in solution (at different pre-conditioning times in the media up to 21 days). Metabolic activity result was normalized to the metabolic activity of untreated HUVEC cells. Scale bars: 100 μm. Results are presented as the mean ± SEM with at least three replicates per group. The significance levels are shown as *p* < 0.05 (*), *p* < 0.01 (**), *p* < 0.001 (***), and *p* < 0.0001(****) for *n* = 3.

The authors confirm that these corrections do not affect the quantification data, data interpretation, or conclusions of the study.

The Royal Society of Chemistry apologises for these errors and any consequent inconvenience to authors and readers.

